# Supplementary material for: Conformational dynamics of free and membrane-bound human Hsp70 in model cytosolic and endo-lysosomal environments
Source: Commun Biol. 2021 Dec 7;4:1369. doi: 10.1038/s42003-021-02892-7 (PMC8651726; doi:10.1038/s42003-021-02892-7)
Supplement: Supplementary file 2 — Supplementary Information [file 42003_2021_2892_MOESM2_ESM.pdf]

Supplementary information

Supplementary note 1: Buffer preparation

Particular efforts were made for optimizing the buffer preparation; therefore, pH measurements were carefully performed. In Supplementary data 1, we list the pH of the undeuterated buffers, the pH_{read} of the deuterated buffers and the calculated pD at the indicated deuterium content. The deuterium content at which the pH_{read} was measured and pD estimated (by adding a factor $0.4xD_{frac}$ to the pH_{read}) was the one used in the corresponding HDX labeling solution.

Supplementary note 2. Liposome measurement

From the liposome preparation, monodisperse suspensions of large unilamellar vesicles (LUVs) incorporating or not BMP, respectively hereinafter referred as BMP^+ and BMP^-, were obtained. Before extrusions, both suspensions had a total lipid concentration at 3.8 mM, with BMP at 0.76 mM in the LUVs BMP^+, substituted with additional PC in the LUVs BMP^-. No efforts were made to estimate the amount of liposomes recovered after extrusion. The results of the Dynamic Light Scattering analysis are shown in Supplementary data 2. For all preparations, polydispersity index (PdI) was <0.08, indicating nearly monodisperse samples, and liposomes presented a comparable mean diameter, within the range of 110-130 nm.

Supplementary note 3. Quench buffer selection

The sequence coverage, *i.e.* the assignment of the generated peptic peptides along the protein sequence, was maximized by merging the identifications resulting from MS/MS spectra of the digested proteins obtained by data dependent and independent acquisition mode. In total, 311

peptides showed to have the requirements for a confident identification, yielding to 98.6% of sequence coverage and 8.05 of average redundancy. Subsequently, in order to evaluate the impact of the four lipids on pepsin cleavage, chromatographic separation and in-source ionization, His6-Hsp70 WT was diluted alone or with the LUVs BMP$^+$ (protein: lipid ratio 1:660) into the aqueous buffer at pH 4.5. The mixtures were added with ice-cold quench buffer (1:1) of three different types: 300 mM phosphate buffer, 300 mM phosphate buffer with 6 M GndHCl or 6 M Urea, all the three adjusted at pH 2.3. Samples were analyzed by LC-MS as described for deuterated samples and peptide signal intensities were inspected on DynamX 3.0, averaged and compared. No shift in peptide retention times was detected, while a general decrease in signal intensities, thus number of peptides detected with S/N>3, was observed in the presence of LUVs BMP$^+$, as summarized in Supplementary data 3. As shown in Fig. S1 and Supplementary data 3, when LUVs BMP$^+$ were not present in the sample, phosphate buffer without additives allowed the highest average peptide signal intensity (and the largest number of peptides detected), whereas the addition of Urea and GndHCl caused 20% and 30% intensity reduction, respectively. Nevertheless, our results highlighted that the presence of liposomes with BMP severely impacted results for the 300 mM phosphate buffer quench buffer, with only 5.3% of average signal intensity retained. Interestingly, the addition of chaotropic agents partially mitigated this detrimental effect of BMP, with quench buffers with Urea and GndHCl additives respectively allowing to detect peptides with approximately 75% and 55% of average signal intensity compared to peptides under identical quench buffer condition but without BMP. As displayed in the graph (Fig. S1), in the presence of LUVs BMP$^+$ the average signal intensity was respectively 7- and 12-times higher when using the quench buffer added with GndHCl and Urea, compared to 300 mM phosphate buffer. Taken together, our data suggest the use of 300 mM phosphate buffer with 6 M Urea (pH 2.3) as buffer for quenching the HDX reaction; therefore, we worked with this buffer to assess the peptide signal intensities after LC-MS analysis of a protein sample containing LUVs BMP$^-$, in the attempt to identify the lipid species responsible for the effect. Our data demonstrated that the phospholipid BMP was specifically responsible for decreasing the

peptide signals, since in the presence of LUVs BMP^- the average intensities did not decrease (Fig. S1). This phenomenon was confirmed also for a model protein (hemoglobin) used in the laboratory (data not shown), thus it is not protein specific. The cause of intensity loss induced by BMP was not investigated further. We hypothesize that this phenomenon is due to the negatively-charged phospholipid impairing the pepsin enzymatic activity or binding to column stationary-phases to impart partial cation-exchange properties and thus deplete positively-charged peptides. Urea and GndHCl added to the quench buffer may be able to interact with the anionic head of the lipid, shielding BMP interaction with the enzyme or column stationary phases and mitigating the undesirable properties of BMP.

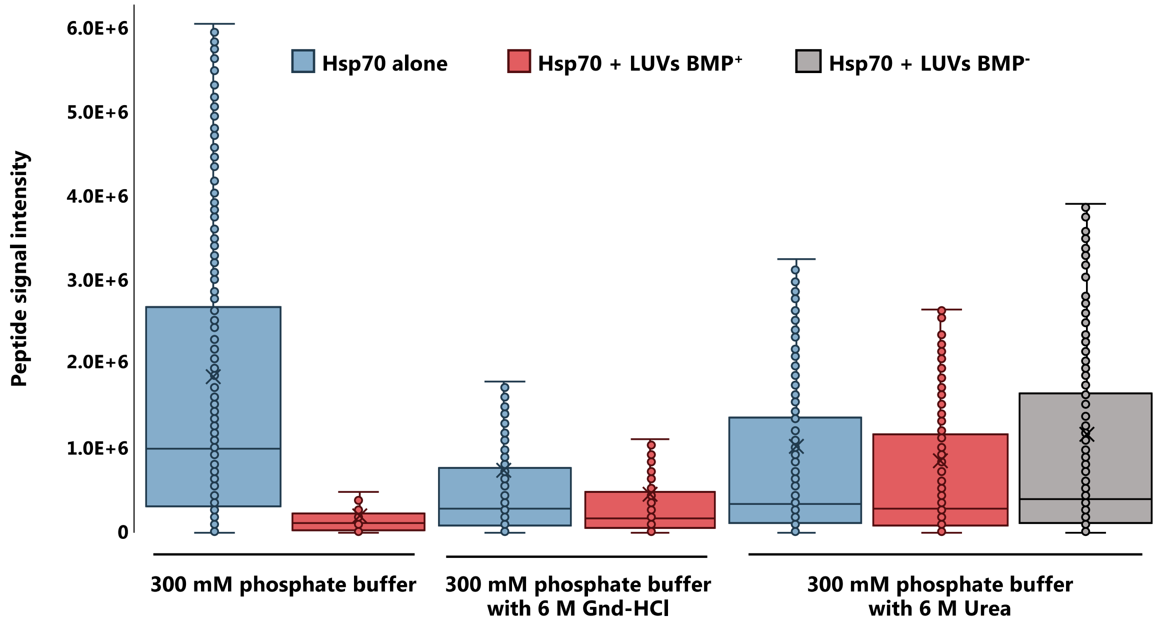

Figure S1. Impact of lipids and quench buffers on peptide signal intensity. Peptide signal intensities in presence or absence of lipids and under different quench buffer conditions are reported.

Supplementary note 4. Conformational impact of BMP on Hsp70 in molten globule conformation at pH 4.5 and 5

Our first attempt to probe by HDX-MS the BMP-induced conformational changes on Hsp70 under lysosomal acidic pH were conducted in acetate buffer at pH 4.5, aiming to replicate the experimental

conditions applied by Kirkegaard *et al*[1]. His[6]-Hsp70 WT was incubated with freshly-made LUVs BMP$^+$ and BMP$^-$ (protein: lipid ratio 1:660) or with the undeuterated HDX buffer for 20 min, in order to allow binding to equilibrate prior to the labeling. HDX was then initiated by 6.8-fold dilution into the corresponding deuterated buffer at room temperature, resulting in 84% deuterium content in the reaction mixture. At various time intervals (1 min, 10 min, 100 min, and 1000 min), an aliquot was withdrawn and quenched 1:1 with ice-cold phosphate buffer with 6M Urea (final pH_{read} 2.3). After quenching, samples were immediately frozen and kept at -80°C until LC-MS analysis. The labeling for 100 min was performed one additional time, producing two technical replicates for those time points. Maximally labeled controls were also included in the experiment.

Upon deuteration, protein backbone amides are able to exchange hydrogens with deuterium atoms during the quick unfolding-refolding events occurring for proteins in solution, according to their chemical exchange constant (k_{ch}), as described by the following equilibrium reactions:

$$NH_{cl} \underset{k_{cl}}{\overset{k_{op}}{\rightleftarrows}} NH_{op} \underset{D_2O}{\overset{k_{ch}}{\rightarrow}} ND_{op} \underset{k_{op}}{\overset{k_{cl}}{\rightleftarrows}} ND_{cl}$$

where N represents a backbone amide covalently bond to either hydrogen (H) or deuterium (D), and k_{op} and k_{cl} describe the constants of the transient and reversible opening and closing events, respectively. The exchange rate of a given amide is thus correlated to its conformational stability, depending on the extent and flexibility of the hydrogen bonding network that it engages in. Upon binding, peptides encompassing the backbone amides directly or indirectly involved in the interaction commonly show reduced HDX as a consequence of the conformational stabilization. This is referred as a type 1 or canonical scenario[2]. Unpredictably, our result highlighted a scenario that drastically differed from the canonical one. At pH 4.5 and 5, several peptides spanning the protein region from residue 10 to 544, corresponding to the NBD, the linker, the subdomain SBDβ and the alpha-helix A and B comprised in the SBDα, were characterized by bimodal-shaped isotopic envelops in the state 'protein only' and 'protein + LUVs BMP^{-}' (Fig. S2-S5). Bimodal-shaped isotopic envelopes

reflect the existence in solution of two protein populations that do not interconvert or do so only very slowly on the HDX timescale. For each of these populations, the HDX can be measured separately on the m/z scale at certain time intervals. The bimodal envelopes were characterized by a low- and a high-mass envelope interchanging in intensity over time in favor to the latter. At pH 4.5 the high-mass envelopes always appeared to have higher relative intensities compared to those appearing at pH 5. The deuterium incorporation calculated for the high-mass envelope of for every peptide was lower than the maximally labeled sample, except for the two overlapping peptides 69-86 and 69-118 (that include Trp90). Analogous results were obtained for the analysis of Hsp70 WT, thus this behavior was not specific to His^6-Hsp70 WT.

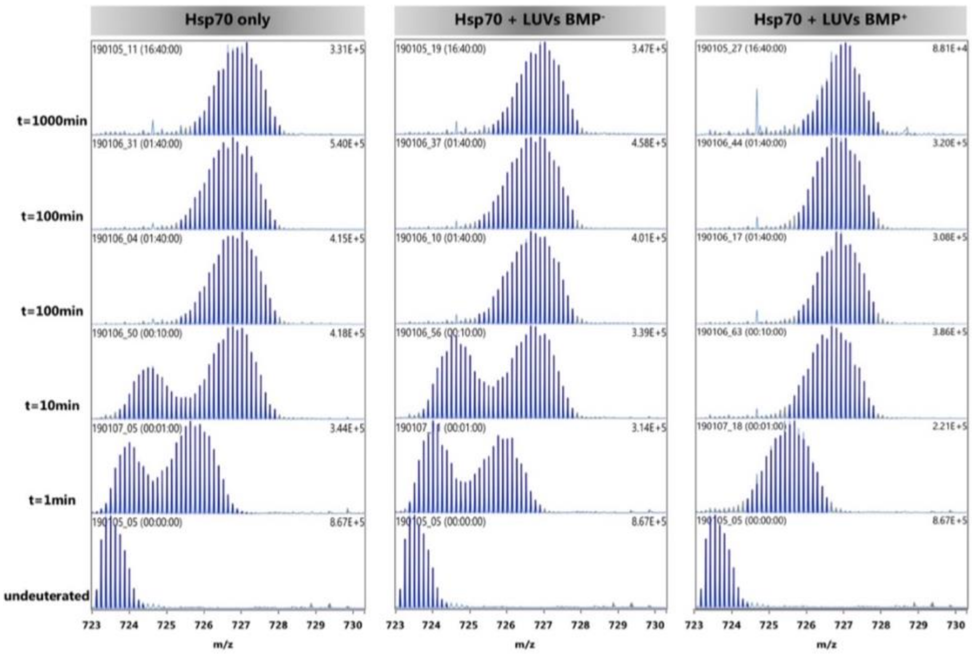

Figure S2. Bimodality of the NBD of Hsp70 at pH 4.5 (BMP enhances unfolding). Mass spectra of peptide 69-118 (+8) in the state "Hsp70 only", "Hsp70 + LUVs BMP^{-}" and "Hsp70 + LUVs BMP^{+}". After 10 min of deuteration, Hsp70 is completely unfolded when incubated with BMP; conversely, the bimodal isotope envelope appears in the states without BMP. At 1 min, the incomplete deuteration at pH 4.5 shifts the isotopes envelopes at lower masses.

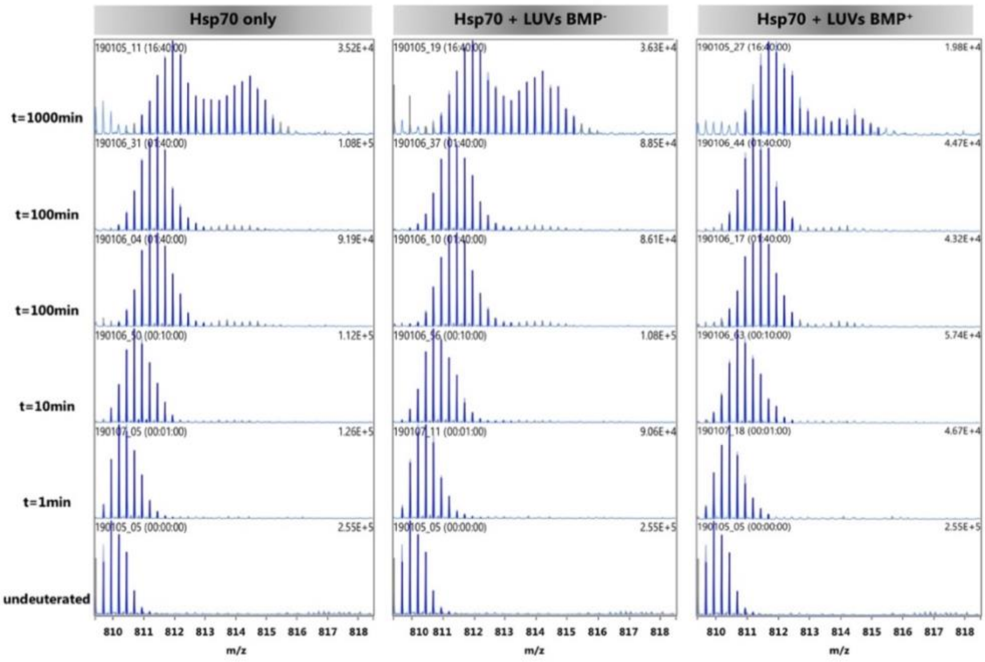

Figure S3. Bimodality of the SBD of Hsp70 at pH 4.5 (BMP enhances the folded population). Mass spectra of peptide 411-439 (+4) in the state "Hsp70 only", "Hsp70 + LUVs BMP^{-}" and "Hsp70 + LUVs BMP^{+}". At the time point 1000 min, the high-mass envelope, corresponding to the unfolded population of Hsp70, is lower in intensity when the protein is incubated with BMP compared to the states without BMP.

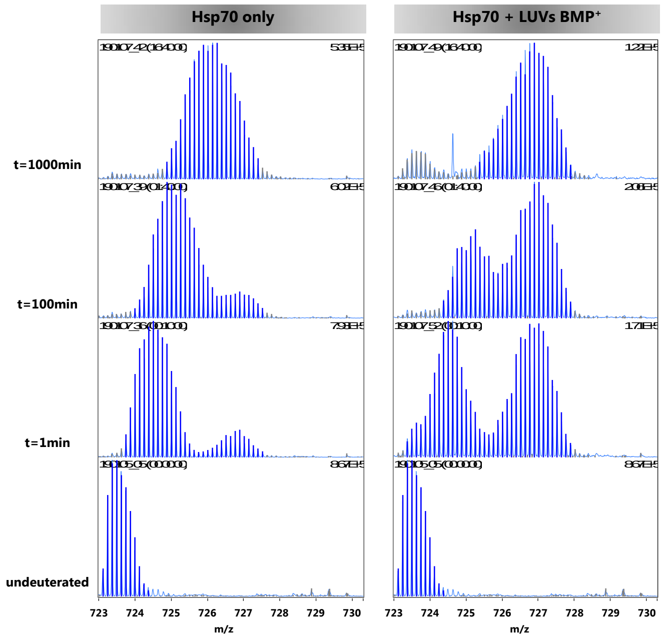

Figure S4. Bimodality of the NBD of Hsp70 at pH 5 (BMP favors the unfolding). Mass spectra of peptide 69-118 (+8) in the state “Hsp70 only”, and “Hsp70 + LUVs BMP⁺”. After 10 min and 100min of deuteration, the relative intensity of the high-mass envelopes is higher in the presence of BMP compared to protein only. An EXx-like kinetics is visible in the mass spectra.

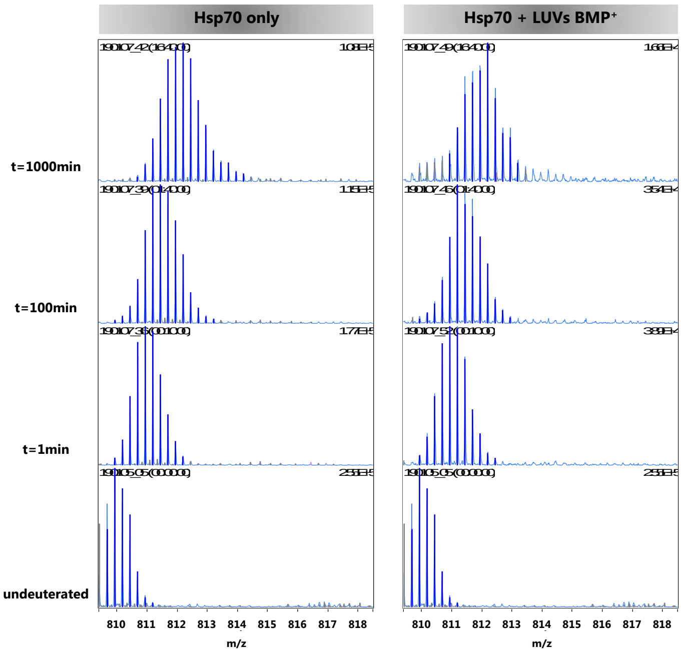

Figure S5. BMP induces protection toward HDX in the SBD at pH 5. Mass spectra of peptide 411-439 (+4) in the state “Hsp70 only”, and “Hsp70 + LUVs BMP+”. At the time point 1000 min, the peptide shows lower deuterium incorporation when Hsp70 is incubated with BMP compared to the states without BMP.

Such atypical behavior of the protein alone or incubated with LUVs BMP^- might be due to EX1 (also called correlated exchange) or EXx kinetics, the letter arising from a mixed effect of EX1/EX2 regimes[3], widespread along most of the protein structure. The exchange rate of a given protein region follows an EX1 kinetics when its $k_{cl} << k_{ch}$, thus the chemical exchange rate (k_{ch}) is high enough to afford labeling an entire group of amides during a single opening event. Nevertheless, for stable proteins (where $k_{cl} >> k_{op}$)[3], such a conformational condition is rarely observable at physiological pH and let alone under acidic conditions, given the minimal value of the k_{ch} at low pH. Conversely, we hypothesized that the HDX of Hsp70 measured at pH 4.5 and 5 was the result of Hsp70 undergoing irreversible unfolding during the time-frame of the HDX experiment. To test this hypothesis, a pulse HDX experiment[4,5] was conducted on His^6-Hsp70 WT diluted in the undeuterated buffer at pH 4.5. Aliquots were withdrawn at various time intervals (0 min, 40 min, 90 min, 240 min, 490 min and 990 min) and pulse labeled for 10min under temperature-controlled conditions (25°C) by 6.8-fold dilution into the deuterated buffer, resulting in 84% deuterium content in the reaction mixture. The pulse was set at 10min, given the low k_{ch} value at this pH. The reaction was quenched 1:1 by adding ice-cold 300 mM phosphate buffer with 6 M Urea (final pH_{read} 2.3) and samples were kept frozen at -80°C until LC-MS analysis. No technical replicates were performed. Maximally labeled controls were also included in the experiment. Our results showed varying deuterium incorporation over the different pulses in the above-mentioned regions (Fig. S6 and S7), whereas constant HDX was rather measured for the region spanning residues 549-638 (Fig. S8). Additionally, MS spectra of several long peptides (>11 residues) allowed to distinguish on the m/z range two isotopic envelopes, with the intensity of the low-mass envelope (corresponding to a more folded population) and the high-mass population (corresponding to a less folded population) respectively decreasing and increasing over time. Our data clearly indicated the lack of stability of Hsp70 under this pH condition, highlighting a gradual unfolding of the whole protein structure, except for the stable alpha-helix C, D, and E of the SBDα and its disordered C-terminal domain. The maximally labeled peptides, which show the deuterium incorporation of a completely unfolded structure, revealed higher centroid mass

compared to the high mass envelopes, excepts for peptides spanning the region 69-118, as observed in the continuous labeling. These results indicate that large parts of Hsp70 retain some secondary structure even after unfolding, while the region that comprise residues 70-118 is completely unfolded. The pulse-labeling experiment also provided information on the unfolding rate of the two domains, with the NBD appearing to unfold faster compared to the SBD. In fact, at 1000 min the SBD peptides still manifested the presence of the low-mass folded population (Fig. S7), already disappeared or present at low intensity at 50 min in the NBD (Fig. S6).

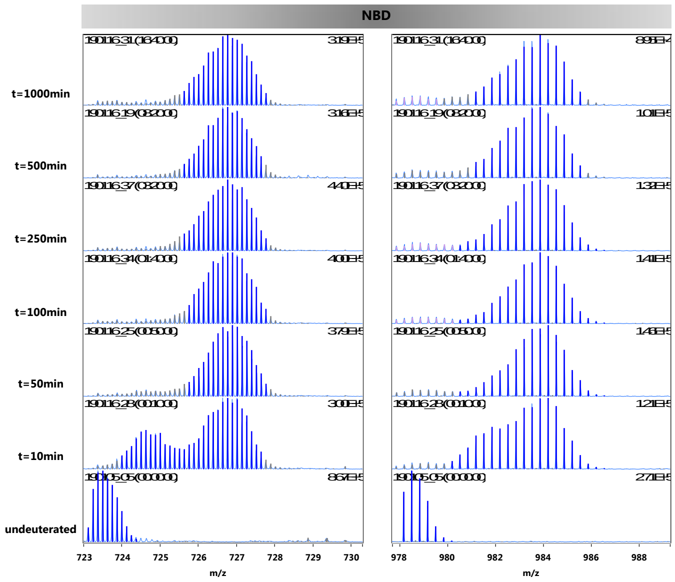

Figure S6. Unfolding over time of the NBD detected by pulse-labelling experiment. Peptide 69-118 (+8) (left panel) and 42-68 (+3) (right panel) show bimodal isotope envelope the time interval 0-10min, which is no longer visible in subsequent time intervals. This behavior indicates a fast unfolding rate.

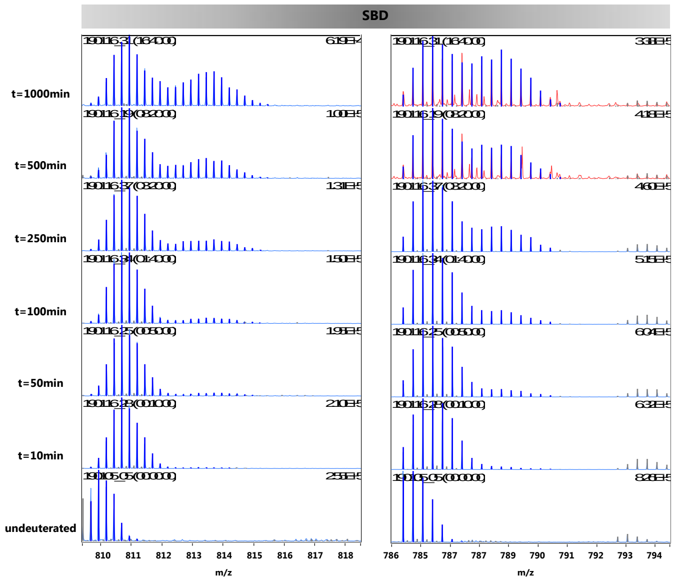

Figure S7. Unfolding over time of the SBD detected by pulse-labelling experiment. Peptide 411-439 (+2) (left panel) and 440-459 (+3) (right panel) show bimodal isotope envelope at any every time interval, with the high mass envelope (corresponding to the unfolded population) increasing in intensity over time. This behavior indicates a slower unfolding rate compared to the NBD.

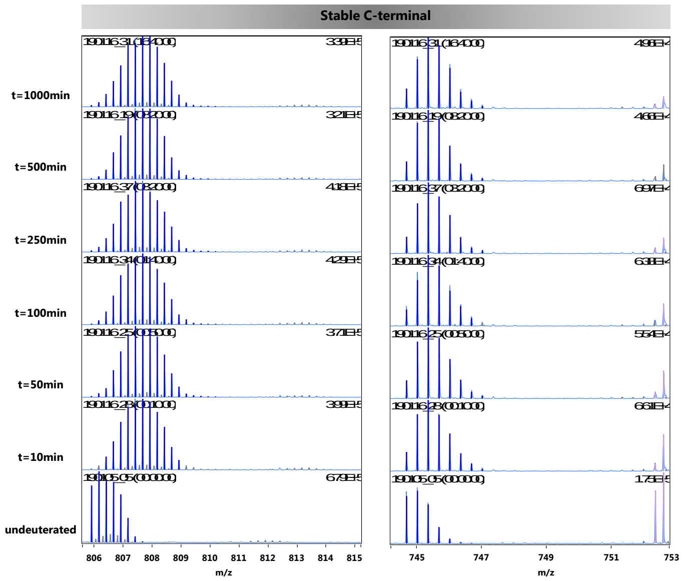

Figure S8. Stability of the C-terminus detected by pulse labelling experiment. Peptide 582-610 (+4) and 478-486 (+1) show unimodal HDX behavior and identical deuterium incorporation over the various pulses.

Both experimental approaches (continuous and pulse labeling) suggest the existence of a molten globule (MG) state for Hsp70 in lysosomes at pH≤5. Molten globules are non-native protein states characterized by a substantial degree of secondary structure and nearly total absence of a native tertiary fold[6,7]. Such partially unstructured states are implicated in various protein functions, including protein translocation through membranes, oligomerization and even catalysis[7]. Our HDX results highlight a slow but spontaneous transition into molten globule state upon dilution of Hsp70 into the acetic buffer, which mimics the acidic lumen of mature lysosomes (pH 4.5-5). The pulse HDX experiment indicates the presence of 1) a folded and 2) an unfolded molten globule conformer, with no transitional states. In most instance, the unfolded conformer shows more protection to HDX than the maximally labeled control (which represents a fully unfolded species), corroborating with the existence of a MG state that, by definition, retains elements of secondary structure. Our results thus indicate that Hsp70 transitions into a MG conformation upon translocation into the lysosomes. MG states are generally induced by very low pH (<2) and/or the addition of denaturing agents, such as guanidinium chloride or alcohols. We thus exclude the possibility of an unwanted artificial induction of a molten globule. Conversely, we consider the observed effect as reflecting an *in vivo* tendency of Hsp70 to assume a MG conformation upon translocation into the lysosomal environment. Our

hypothesis is supported by the fact that the MG conformation is not a rare phenomenon for proteins translocating into the lysosomal compartment, and it has been proved for other species[8-14].

We can thus conclude that the continuous labeling was, in fact, a dynamic labeling experiment, where the equilibrium reaction of the structurally unstable protein, probed by HDX in a conformer-specific manner, can be described as follows:

$$NH_{Native} \underset{k_{refolding}}{\overset{k_{unfolding}}{\rightleftarrows}} NH_{MG-cl} \underset{k_{cl}}{\overset{k_{op}}{\rightleftarrows}} NH_{MG-op} \underset{D_2O}{\overset{k_{ch}}{\rightarrow}} ND_{MG}$$

where $k_{\mathrm{unfolding}}$ and $k_{\mathrm{refolding}}$ indicates the rate of the unfolding and refolding event, while k_{op} and k_{cl} describe respectively the constants of the transient and reversible opening and closing events occurring during the spontaneous fluctuations of the MG secondary structure elements. As demonstrated by our pulse HDX, Hsp70 unfolds in a minute/hour time scale, while the breathing motions of the MG occurs in the sub-second time scale. Therefore, since the limiting step toward the amide deuteration is represented by the protein unfolding ($k_{\mathrm{unfolding}} << k_{\mathrm{op}}/k_{\mathrm{cl}}$), the above equilibrium can be simplified as follows:

$$NH_{Native} \underset{k_{refolding}}{\overset{k_{unfolding}}{\rightleftarrows}} NH_{MG} \underset{D_2O}{\overset{k_{ch}}{\rightarrow}} ND_{MG}$$

Contrary to what occur for stable proteins where $k_{\mathrm{cl}} >> k_{\mathrm{op}}$, the unfolding-refolding equilibrium of Hsp70 at pH 4.5 is shifted toward the unfolded state, leading to $k_{\mathrm{unfolding}} >> k_{\mathrm{refolding}}$. Therefore, the k_{HX} can be elaborated by the following equation:

(eq. 1),

$$k_{HX} = \frac{k_{unfolding} \times k_{ch}}{k_{unfolding} + k_{ch}}$$

Given $k_{\mathrm{ch}} >> k_{\mathrm{unfolding}}$, the equation 8 simplifies into equation 9:

(eq. 2),

$$k_{HX} = k_{unfolding}$$

that compares to the equation describing the EX1 regime for folded proteins[3]. In parallel, the folded conformer follows the standard equilibrium of folded proteins in solution, which is assumed to uniquely manifest as EX2 regime at pH 4.5-5, generating the bimodal behaviors observed for Hsp70 alone and incubated with LUVs BMP^-. From equation 9, we can therefore deduce that the measured HDX of Hsp70 at pH 4.5 also provides insights on the MG-transition rate of the different protein regions. HDX-MS has already been used by other groups to investigate molten globule states behavior in solution[15-18], although the experiments failed the detection of different protein conformers, without applying denaturing agents that artificially induce EX1 exchange regime. Indeed, MG formation is favored under acidic buffer conditions, where the intrinsic exchange rates of unprotected amides is dramatically decreased, leading HDX to typically follow the so-called uncorrelated regime (EX2 kinetics). Despite that, our HDX analysis allowed distinct observation of the two protein states, native and MG, under conditions which fully reproduces the protein native environment, due to the low unfolding rate.

Remarkably, the presence of LUVs BMP^+ was observed to accelerate the unfolding rate of the NBD (Fig. S2 and S4) and to reduce it for the SBD (Fig. S3 and S5). More in details, for the state 'protein + LUVs BMP^+' peptides generated from the NBD showed either higher intensity in the high-mass population compared to 'protein only' and 'protein + LUVs BMP^-' or the presence of the high-mass isotopic envelope only, with the exception of regions comprising residues 19-41, 182-198 and 295-305 where the BMP enhanced the more folded structure. Conversely, peptides generated upon digestion of the SBD showed opposite effect, with the low-mass isotopic envelope higher in intensity compared to 'protein only' and 'protein + LUVs BMP^-'. BMP did not induce any effect at the level of the linker between the two domains and in the stable regions of the SBD. Fig. S9 summarizes the BMP effect on the unfolding rate and the results of the pulse labeling.

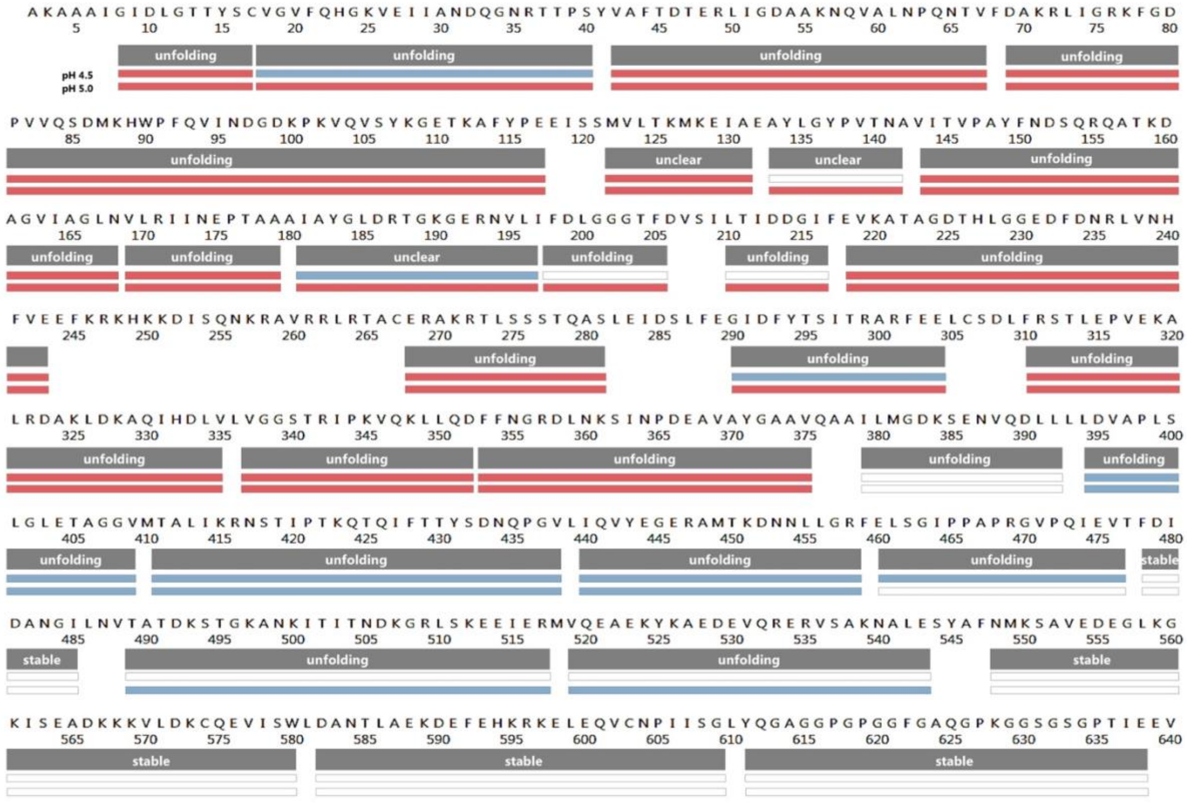

Fig. S9. BMP-induced conformational changes at pH 4.5 and 5. The grey square describes the behavior of the region at pH 4.5 highlighted by the pulse-labelling experiment. The bars below the grey square indicate the BMP-induced conformational changes at pH 4.5 (upper) and 5 (down). Red color indicates that BMP enhances the unfolded population or induces and increased HDX. Blue color indicates that BMP favors the folded population or protects from HDX. White color indicates no differences in HDX. The HDX of the following peptides was considered for this analysis, selecting those with the higher number of residues to better elucidate the local kinetic behavior: 9-17, 18-41, 42-68, 69-86, 69-118, 122-132, 134-142, 143-169, 168-180, 181-198, 197-206, 220-217, 218-244, 269-282, 290-296, 294-305, 310-336, 337-353, 353-376, 379-393, 394-410, 411-439, 440-459, 460-477, 478-486, 489-518, 519-544, 530-544, 548-576, 565-581, 582-610, 611-638.

These observations are hallmarks of a non-canonical but undeniably different HDX scenario in presence of BMP, indicating a $k_{unfolding}$ significantly impacted when Hsp70 is incubated with the anionic phospholipid. We hypothesize that at pH 4.5 the anionic phospholipid assists the MG transition of the NBD by subtracting the unfolded species NH_{MG} as bound species $NH_{MG\text{-}bound}$ from the equilibrium with the folded conformer. Considering the lack of this effect with LUVs BMP^-, we exclude that the association could involve BMP acyl chains (18:1), as these are identical to one of the two fatty acid

tails of POPC (18:1 and 16:0). We rather believe that the interaction can happen through the anionic head group of BMP engaging in electrostatic interactions with the exposed basic residues after unfolding, an interaction that cannot occur through the positively charged head of PC. The HDX of a protein in solution is influenced by its hydrogen bonding network with the solvent and its solvent accessibility. The lack of protection to HDX in the MG-bound state suggests that Hsp70 binds to the lysosomal membrane by electrostatic interactions with side chains, in a fashion that has only immeasurable minor effects on the backbone amides, which remain solvent-exposed. This scenario, named type 0 scenario[2], cannot be described by HDX-MS experiments[19], as side chain deuterium atoms back-exchange into hydrogens in the millisecond time scale under quench conditions. A peripheral association through the side chains is likely to be the driving mechanism of the interaction of the molten globular Hsp70 to lysosomal membranes at pH 4.5-5.

Supplementary note 5. Hsp70 remains folded at pH equal or higher than 5.3

Results acquired at pH 4.5 and 5 revealed non-canonical binding effects of MG Hsp70 to the lysosomes membrane. We thus aimed to investigate the BMP-induced conformational impact on a still-folded chaperone at acidic pH. Moreover, continuous labeling experiments are generally conducted on stable protein structures, whose conformational changes are uniquely generated by the breathing motions of the species in solution. In order to mimic the lysosomal environment, our experiments had to be conducted under acidic conditions hence we aimed to determine the minimal pH enabling protein folding and stability. To achieve this, deuterium exchange of Hsp70 was evaluated in acetic buffers with reduced acidity. His6-Hsp70 WT was diluted in undeuterated buffers at pH 5.3 and 5.5. Aliquots were withdrawn at 0, 8 min, 98 min, and 998 min and pulse labeled for 2 min under temperature-controlled conditions (25°C) by 4.6-fold dilution into the corresponding deuterated buffer, yielding to 78% deuterium content in the reaction mixture. The reaction was quenched 1:1 by adding ice-cold 300 mM phosphate buffer with 6 M Urea (final pH_{read} 2.3) and samples were kept frozen at -80°C until

LC-MS analysis. No technical replicates were performed. Maximally labeled controls were also included in the experiment. For both pH tested, peptides showed identical HDX upon deuteration of Hsp70 incubated for 2 min, 10 min, 100 min and 1000 min in the aqueous buffers, and the bimodal isotope distributions did not manifest at any time interval (Fig. S10 and S11). These data indicated the absence of the two populations, a folded and partially-folded, present at pH≤5, which suggests that both domains are stable at pH≥5.3. For this reason, pH 5.3 was selected as a condition to probe Hsp70 binding to lipids (see main text). At pH 5.3, Hsp70 thus does not undergo the transition into MG, and we observe a canonical conformational stabilization (reduced HDX) due to backbone amide protection upon BMP binding (see main text). This suggest that, during the process of maturation from late endosomes (pH 5.5) to lysosomes (pH 4.5-5), Hsp70 adopts different structural conformations and binds to the membrane with different mechanisms, as highlighted for another protein species transitioning into a MG state in the lysosomes[20]. Additionally, these results open to further investigations to assess Hsp70 molten globule conformation in the lysosomes.

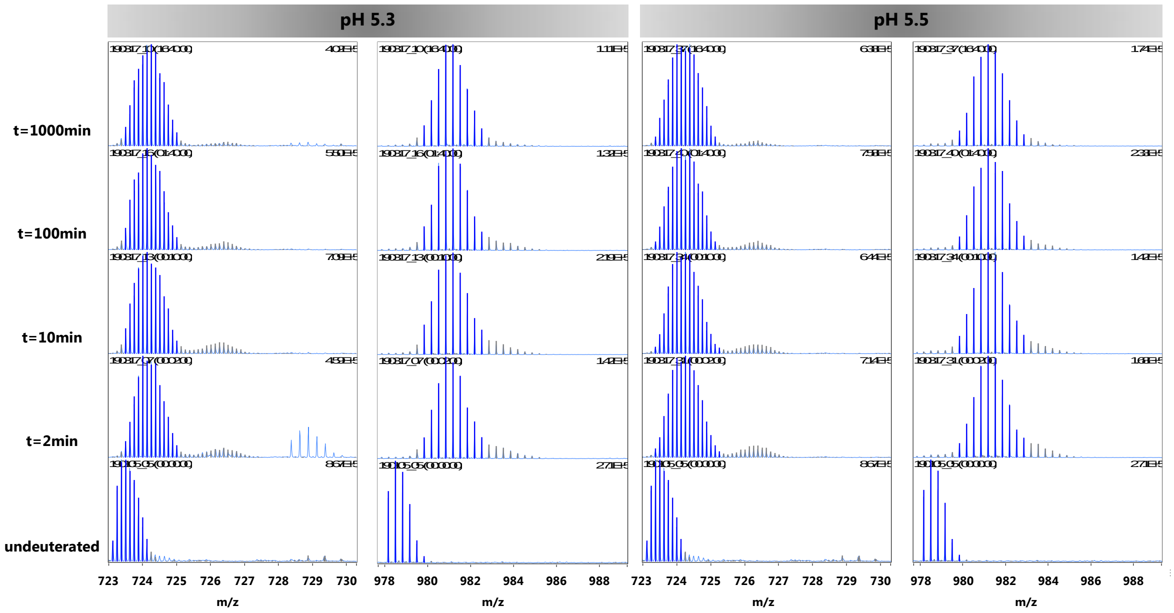

Figure S10. Stabilization of the NBD at pH 5.3 and 5.5. Peptides 69-118 (+8) on the left panel and 42-68 (+3) on the right panel show comparable HDX at any time interval assessed.

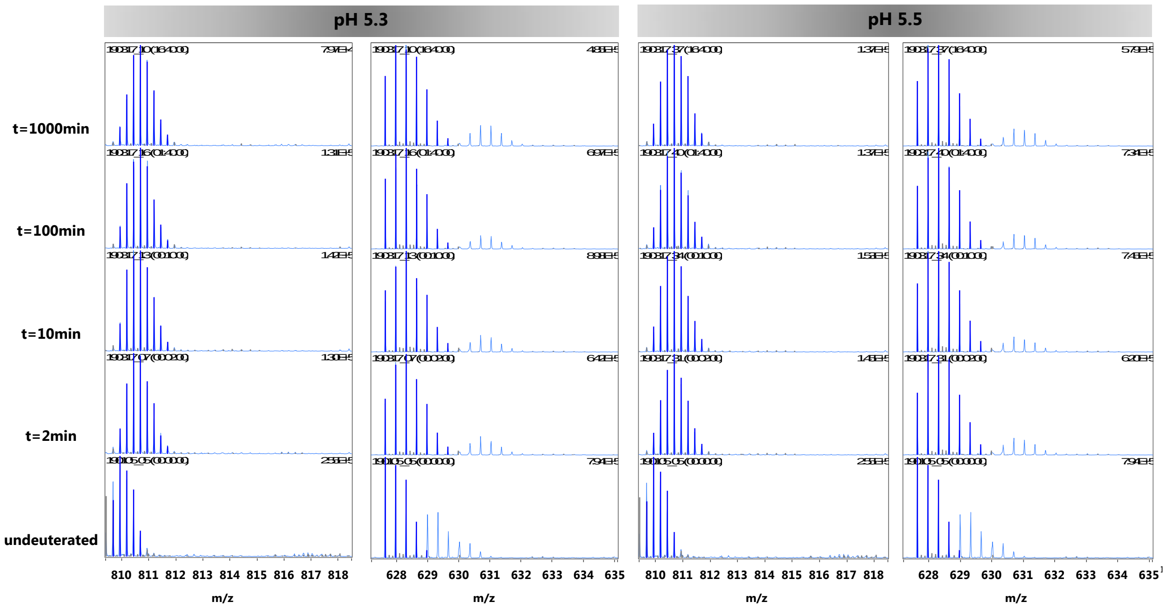

Figure S11. Stabilization of the SBD at pH 5.3 and 5.5. Peptides 411-439 (+4) on the left panel and 440-455 (+3) on the right panel show comparable HDX at any time interval assessed.

Supplementary note 6. Validation of the applied procedure for correcting the effect of pH on k_{ch}

To probe the difference in conformational dynamics of the chaperone in the cytosol and lysosomal compartment, we had to label the protein under different pH conditions. This method implies the calculation of a time-point conversion factor (eq. 3 in the main text) that allows the same number of deuterated amides in a time unit[21,22]. To exclude possible biases induced by experimental factors not considered in the theoretical equation, we empirically evaluated the capability of the two labeling buffers (acetic acid buffer and phosphate buffer) of enabling identical deuteration after time point adjustment . His^6-Hsp70 WT was diluted in solvent A (0.23% formic acid in MilliQ water, pH 2.5) and injected into the UPLC system. Following on-line enzymatic digestion, the generated peptides were manually collected when eluting from the pepsin column. The mobile phase was then evaporated using a SpeedVac (ThermoFisher). The peptides were resuspended into the acetic or phosphate undeuterated buffer and the solutions were let equilibrate for 60 min in a cold room at 4.5°C. HDX was then initiated by 10-fold dilution into the corresponding deuterated buffer at 4.5°C, resulting in 89% deuterium content in the reaction mixture. Samples were quenched 1:1 with ice-cold phosphate

buffer with 6 M Urea (pHread 2.3) after labeling for 5 s (duplicates performed) and 8 min 2 s at pH 7.4 and 5.3, respectively, given the correction factor as 96.38. The whole process was conducted in a cold room at 4.5°C, with all the buffers held at this temperature, to limit full deuteration of every peptide back-bone amide. After quenching, samples were immediately frozen and kept at -80°C until LC-MS analysis. The absence of higher order structure in the peptides cancelled any pH-induced structural effect, allowing us to evaluate the effect of the pH buffer on k_{ch}. For this control experiment, we compared the deuterium uptake of those peptides showing deuterium incorporation at least 0.5 Da lower than the maximally labelled control, since no information can be extrapolated from species showing near complete labeling of deuterated amides. In Supplementary data 4, we list the peptide considered for this analysis and their uptake. As shown in table S4, our data indicate that, by applying the correction factor 96.38 calculated from equation 3, the two deuterated buffers allow identical number of exchanged amides upon time point conversion, ensuring that any difference in deuterium incorporation of Hsp70 at acidic and neutral pH uniquely reflect changes in protein conformation and dynamics.

Supplementary references

1. Kirkegaard, T. et al. Hsp70 stabilizes lysosomes and reverts Niemann–Pick disease-associated lysosomal pathology. *Nature* **463**, 549-553 (2010).
2. Sowole, M. A. & Konermann, L. Effects of protein-ligand interactions on hydrogen/deuterium exchange kinetics: canonical and noncanonical scenarios. *Anal. Chem.* **86**, 6715-6722 (2014).
3. Jensen, P. F. & Rand, K. D. Hydrogen exchange: a sensitive analytical window into protein conformation and dynamics. in *Hydrogen Exchange Mass Spectrometry of Proteins: Fundamentals, Methods and Applications* (ed. Weis, D. D.) 1-17 (John Wiley & Sons, Chichester, West sussex, PO19 8sQ, United Kingdom, 2016).
4. Miranker, A., Robinson, C. V, Radford, S. E., Aplin, R. T. & Dobson, C. M. Detection of transient protein folding populations by mass spectrometry. *Science* **262**, 896-900 (1993).
5. Georgescauld, F. et al. GroEL/ES chaperonin modulates the mechanism and accelerates the rate of TIM-barrel domain folding. *Cell* **157**, 922-934 (2014).
6. Ptitsyn, O. B. How the molten globule became. *Trends Biochem. Sci.* **20**, 376-379 (1995).
7. Dunker, A. K., Brown, C. J., Lawson, J. D., Iakoucheva, L. M. & Obradović, Z. Intrinsic disorder

and protein function. *Biochemistry* **41**, 6573-6582 (2002).

8. Man, P. et al. Defining the interacting regions between apomyoglobin and lipid membrane by hydrogen/deuterium exchange coupled to mass spectrometry. *J. Mol. Biol.* **368**, 464-472 (2007).

9. Man, P. et al. Accessibility changes within diphtheria toxin T domain when in the functional molten globule state, as determined using hydrogen/deuterium exchange measurements. *FEBS J.* **277**, 653-662 (2010).

10. Tseng, T. et al. A molten globule-to-ordered structure transition of Drosophila melanogaster crammer is required for its ability to inhibit cathepsin. *Biochem. J.* **442**, 563-572 (2012).

11. Ji, Z. et al. Reactivity of Apolipoprotein E4 and Amyloid beta Peptide. *J. Biol. Chem.* **281**, 2683-2692 (2006).

12. Van Acker, Z. P., Bretou, M. & Annaert, W. Endo-lysosomal dysregulations and late-onset Alzheimer's disease: impact of genetic risk factors. *Mol. Neurodegener.* **14**, 1-20 (2019).

13. Jerala, R., Zerovnik, E., Kidric, J. & Turk, V. pH-induced conformational transitions of the propeptide of human cathepsin L. A role for a molen globule state in zymogen activation. *J. Biol. Chem.* **273**, 11498-11504 (1998).

14. Bom, A. P. et al. The p53 core domain is a molten globule at low pH: functional implications of a partially unfolded structure. *J. Biol. Chem.* **285**, 2857-2866 (2010).

15. Maier, C. S., Kim, O. H. & Deinzer, M. L. Conformational properties of the A-state of cytochrome c studied by hydrogen/deuterium exchange and electrospray mass spectrometry. *Anal. Biochem.* **252**, 127-135 (1997).

16. Last, A. M., Schulman, B. A., Robinson, C. V & Redfield, C. Probing subtle differences in the hydrogen exchange behavior of variants of the human α-lactalbumin molten globule using mass spectrometry. *J. Mol. Biol.* **311**, 909-919 (2001).

17. Mazon, H., Marcillat, O., Forest, E., Smith, D. L. & Vial, C. Conformational dynamics of the GdmHCl-induced molten globule state of creatine kinase monitored by hydrogen exchange and mass spectrometry. *Biochemistry* **43**, 5045-5054 (2004).

18. Hoerner, J. K., Xiao, H. & Kaltashov, I. A. Structural and dynamic characteristics of a partially folded state of ubiquitin revealed by hydrogen exchange mass spectrometry. *Biochemistry* **44**, 11286-11294 (2005).

19. Ly, T. & Julian, R. R. Using ESI-MS to probe protein structure by site-specific noncovalent attachment of 18-crown-6. *J. Am. Soc. Mass. Spectrom.* **17**, 1209-1215 (2006).

20. Man, P. et al. Accessibility changes within diphtheria toxin T domain upon membrane penetration probed by hydrogen exchange and mass spectrometry. *J. Mol. Biol.* **414**, 123-134 (2011).

21. Coales, S. J. et al. Expansion of time window for mass spectrometric measurement of amide hydrogen/deuterium exchange reactions. *Rapid Commun. Mass Spectrom.* **24**, 3585-3592 (2010).

22. Garcia, N. K., Guttman, M., Ebner, J. L. & Lee, K. K. Dynamic changes during acid-induced activation of influenza hemagglutinin. *Structure* **23**, 665-676 (2015).

Supplementary figures

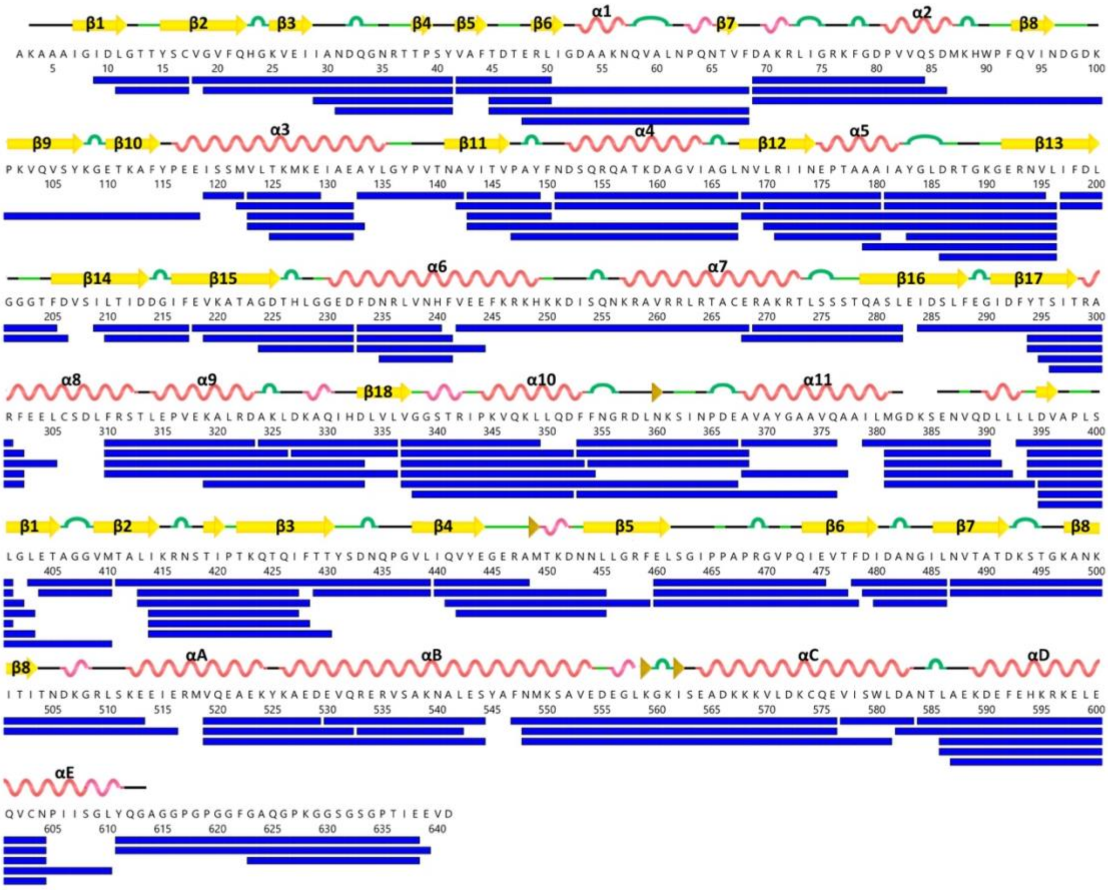

Figure S12. Peptides considered for the analysis of pH comparison and structural comparison between 6His-Hsp70 WT and 6His-Hsp70 W90F are depicted along the protein sequence and its secondary structure elements, as displayed in the sequence view of PDB 1S3X (NBD) and 4PO2 (SBD).

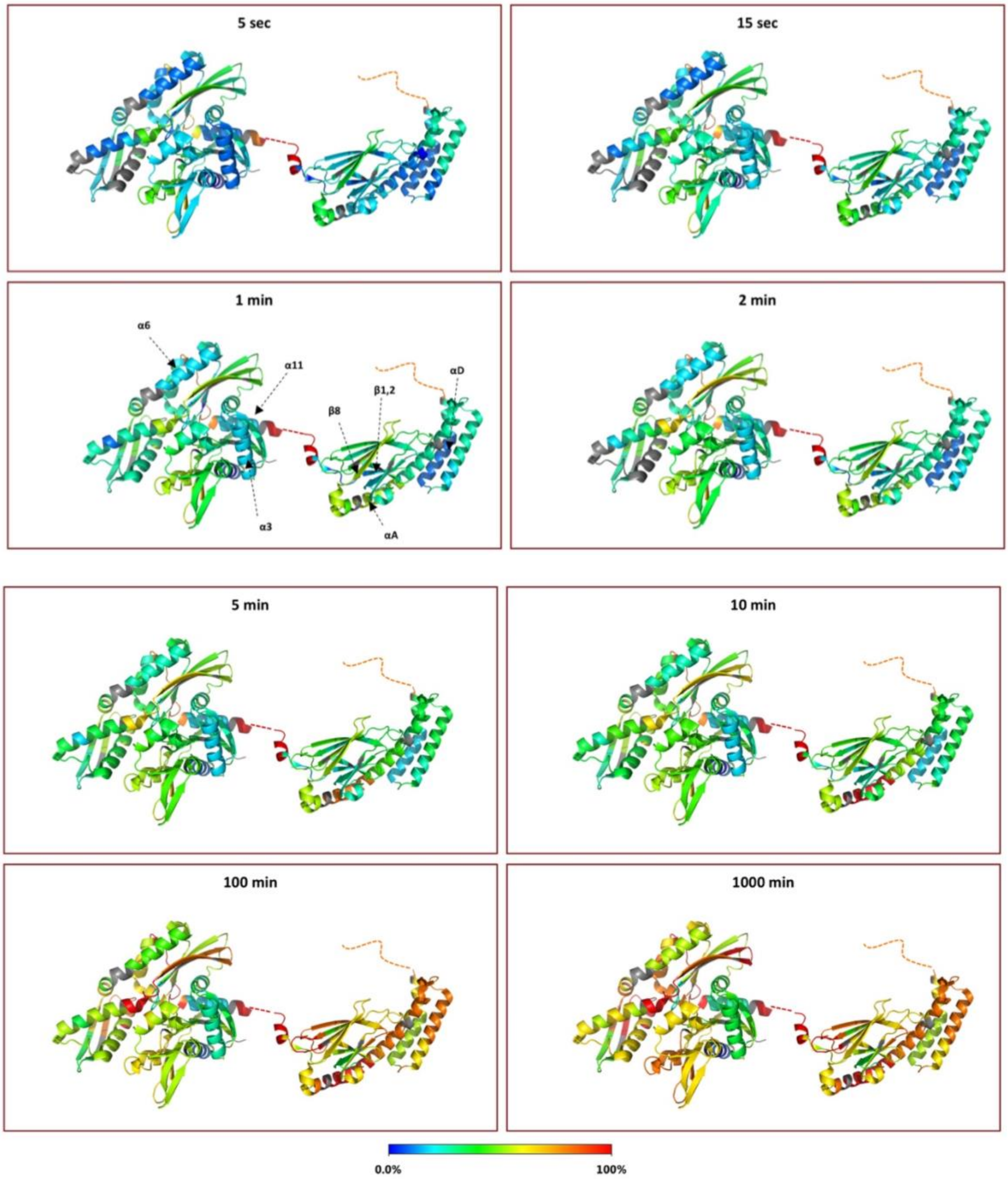

Figure S13. Conformational dynamics of His^6-Hsp70 at pH 7.4. The structural elements of the PDB structures (1S3X and 4PO2) are coloured according to their relative percentage of deuteration, normalized to the maximally labelled control. The dot lines indicate protein portions lacking in the crystalized constructs. Grey colour indicates that no data are available for the segment.

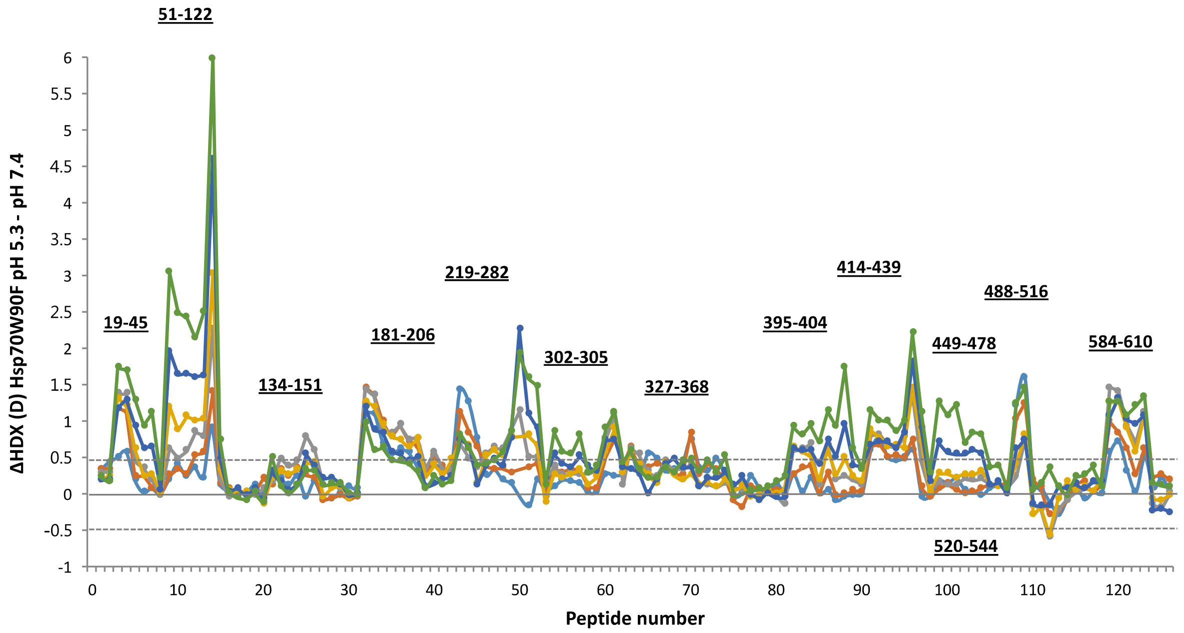

Figure S14. Comparison of HDX of His[6]-Hsp70 W90F at pH 5.3 and 7.4. Difference plot illustrating the difference in HDX between pH 5.4 and 7.4 over the measured time points (cyan line: 5 s, orange line: 15 s, grey line: 1 min, yellow line: 2 min, blue line: 5 min, green line: 10 min). Residues comprising a region with significant difference in HDX are indicated. The peptides are arranged according to their position from N- to C-terminus (Supplementary data 5, table 2). A dotted grey line indicates the 98% CI as a threshold for significance.

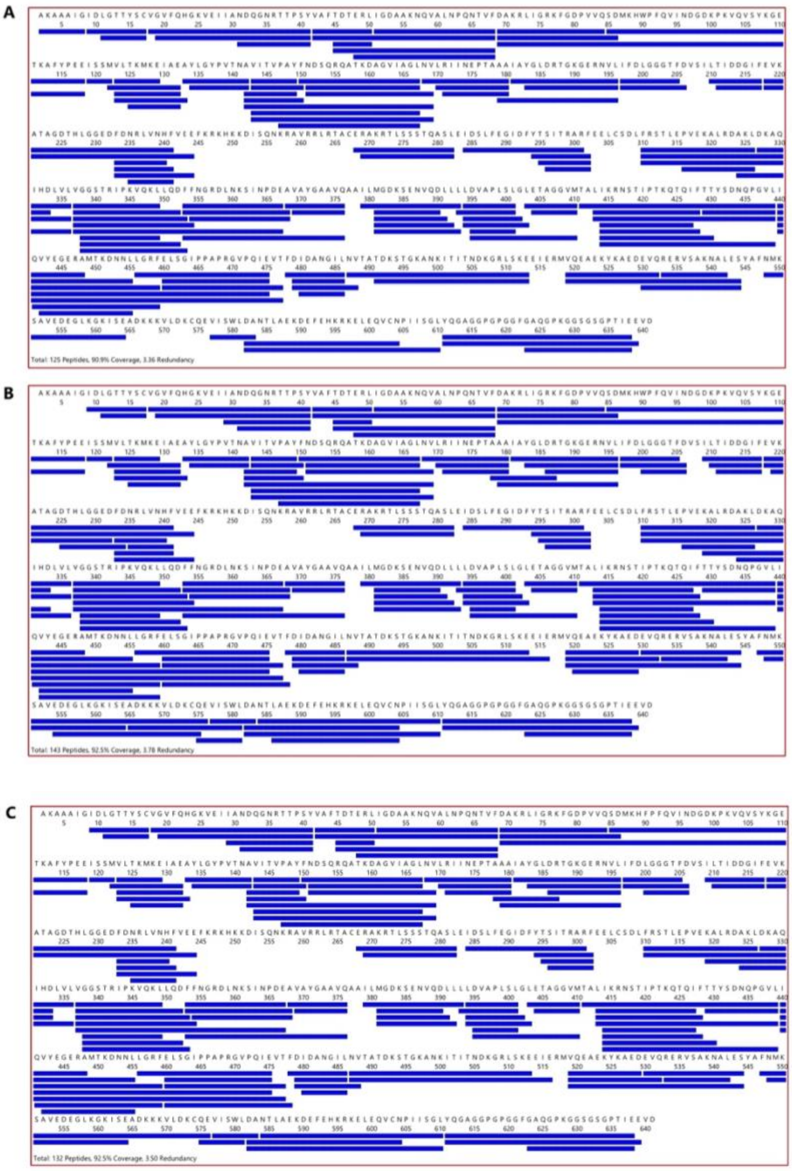

Figure S15. Peptides considered for the analysis of the conformational impact of lipids on A) Hsp70 WT (non-tagged); B) 6His-Hsp70 WT; and C) 6His-Hsp70W90F are depicted along the protein sequence.

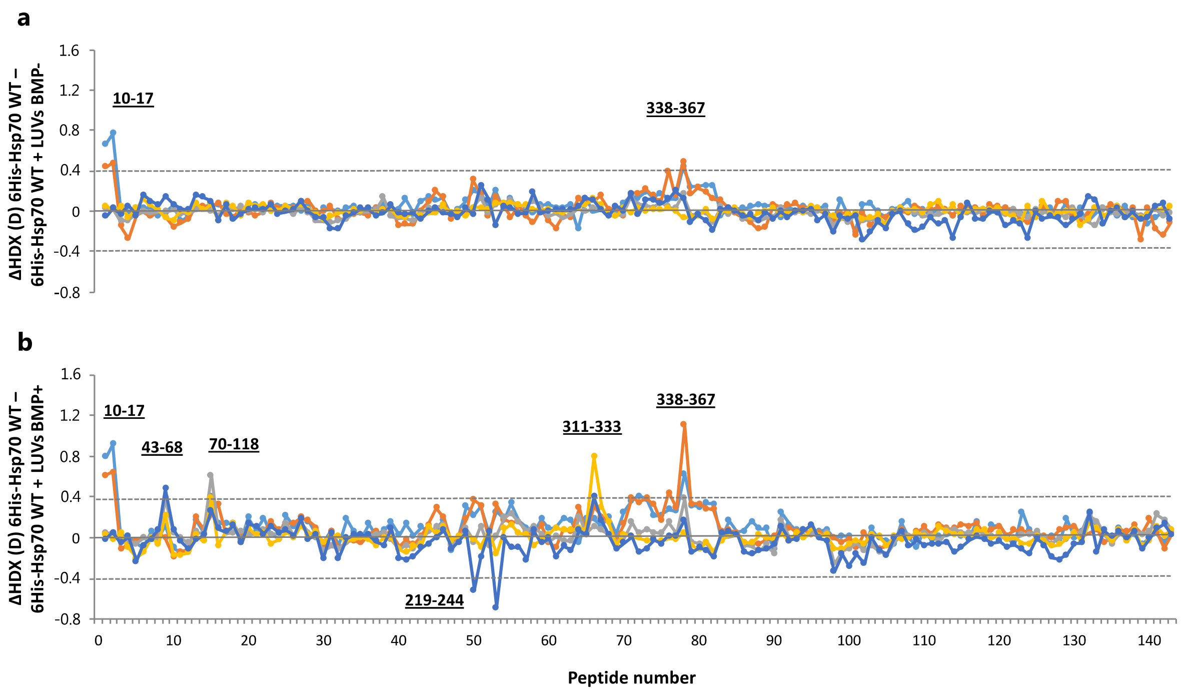

Figure S16. Comparison of HDX of the His^{6}-Hsp70 WT in the presence of liposomes with and without with BMP. Difference plots illustrate difference in HDX over the measured time points between 6His-Hsp70 WT incubated with liposomes without BMP (**a**) and incubated with liposomes and with BMP (**b**). Cyan line indicates 15 s, orange line 1 min, grey line 10 min, yellow line 100 min, blue line 1000 min. Residues comprising a region with significant difference in HDX are indicated. The peptides are arranged according to their position from N- to C-terminus (Supplementary data 5, table 4). Dotted grey lines indicate the 98% CI as a threshold for significance.

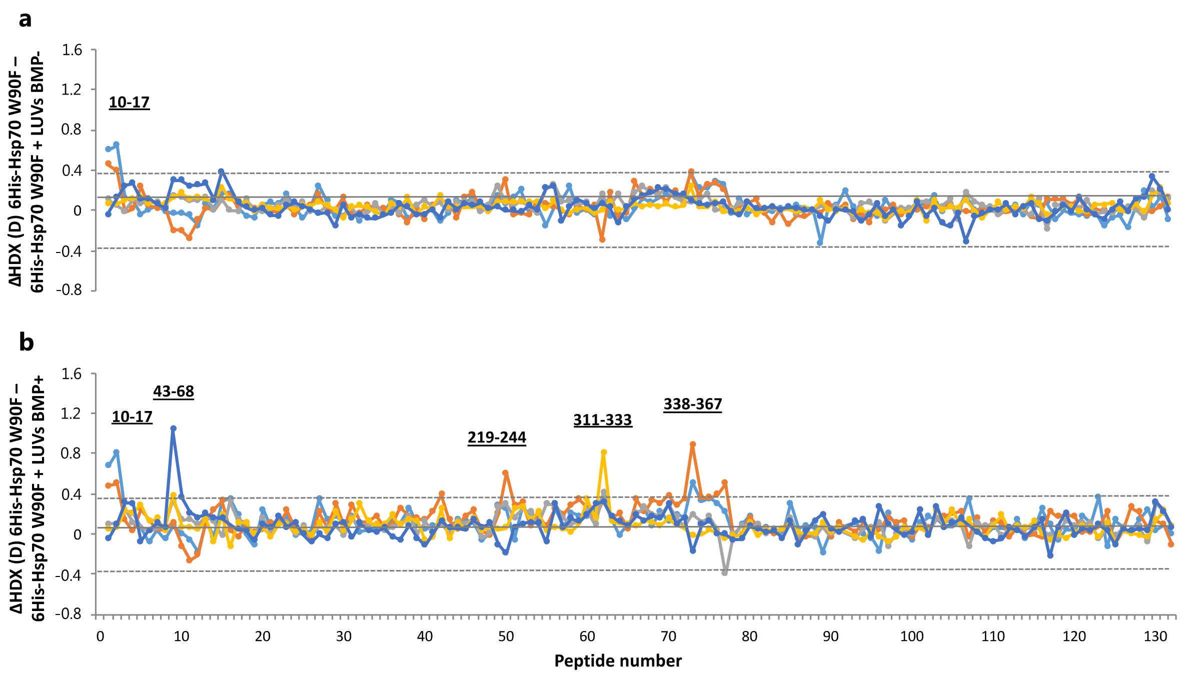

Figure S17. Comparison of HDX of the His^6-Hsp70 W90F in the presence of liposomes with and without with BMP. Difference plots illustrate difference in HDX over the measured time points between 6His-Hsp70 W90F incubated with liposomes without BMP (**a**) and incubated with liposomes and with BMP (**b**). Cyan line indicates 15 s, orange line 1 min, grey line 10 min, yellow line 100 min, blue line 1000 min. Residues comprising a region with significant difference in HDX are indicated. The peptides are arranged according to their position from N- to C-terminus (Supplementary data 5, table 5). Dotted grey lines indicate the 98% CI as a threshold for significance.
