# Supplementary material for: Conformational dynamics of free and membrane-bound human Hsp70 in model cytosolic and endo-lysosomal environments
Source: Commun Biol. 2021 Dec 7;4:1369. doi: 10.1038/s42003-021-02892-7 (PMC8651726; doi:10.1038/s42003-021-02892-7)
Supplement: Supplementary file 3 — Description of Additional Supplementary Files [file 42003_2021_2892_MOESM3_ESM.pdf]

Description of Additional Supplementary Files

File name: Supplementary data 1
Description: pH values of the buffers used for HDX experiments.

File name: Supplementary data 2
Description: Measurements of dimension and dispersion of liposomes.

File name: Supplementary data 3
Description: Sequence coverage and peptides identified with the different quench buffers tested and in presence or absence of liposomes with BMP.

File name: Supplementary data 4
Description: Deuterium uptake values of Hsp70 peptides labelled at pH 7.4 (5 s) and pH 5.3 (8 min 2 s).

File name: Supplementary data 5
Description: HDX summary tables (table 1) and the HDX data tables (table 2: 6His-Hsp70 WT and W90F at pH 7.4 and 5.3; table 3: Hsp70 WT in presence and absence of liposomes; table 4: 6His-Hsp70 WT in presence and absence of liposomes; table 5: 6his-Hsp70 W90F in presence and absence of liposomes).
