# Supplementary material for: Conformational dynamics of free and membrane-bound human Hsp70 in model cytosolic and endo-lysosomal environments
Source: Commun Biol. 2021 Dec 7;4:1369. doi: 10.1038/s42003-021-02892-7 (PMC8651726; doi:10.1038/s42003-021-02892-7)
Supplement: Supplementary file 9 — Reporting Summary [file 42003_2021_2892_MOESM9_ESM.pdf]

nature portfolio

Corresponding author(s): Kasper Dyrberg Rand

Last updated by author(s): Oct 4, 2021

Reporting Summary

Nature Portfolio wishes to improve the reproducibility of the work that we publish. This form provides structure for consistency and transparency in reporting. For further information on Nature Portfolio policies, see our Editorial Policies and the Editorial Policy Checklist.

Statistics

For all statistical analyses, confirm that the following items are present in the figure legend, table legend, main text, or Methods section.

n/a	Confirmed	
☐	☒	The exact sample size (*n*) for each experimental group/condition, given as a discrete number and unit of measurement
☐	☒	A statement on whether measurements were taken from distinct samples or whether the same sample was measured repeatedly
☐	☒	The statistical test(s) used AND whether they are one- or two-sided<br>*Only common tests should be described solely by name; describe more complex techniques in the Methods section.*
☒	☐	A description of all covariates tested
☐	☒	A description of any assumptions or corrections, such as tests of normality and adjustment for multiple comparisons
☐	☒	A full description of the statistical parameters including central tendency (e.g. means) or other basic estimates (e.g. regression coefficient) AND variation (e.g. standard deviation) or associated estimates of uncertainty (e.g. confidence intervals)
☐	☒	For null hypothesis testing, the test statistic (e.g. *F*, *t*, *r*) with confidence intervals, effect sizes, degrees of freedom and *P* value noted<br>*Give P values as exact values whenever suitable.*
☒	☐	For Bayesian analysis, information on the choice of priors and Markov chain Monte Carlo settings
☒	☐	For hierarchical and complex designs, identification of the appropriate level for tests and full reporting of outcomes
☒	☐	Estimates of effect sizes (e.g. Cohen's *d*, Pearson's *r*), indicating how they were calculated

Our web collection on statistics for biologists contains articles on many of the points above.

Software and code

Policy information about availability of computer code

Data collection	MassLynx (v. 4.1, Waters) was used for controlling the Synapt G2-Si mass spectrometer and for the acquisition pf MS and MS-MS data
Data analysis	ProteinLynx Global Server (v. 3.0, Waters) was used for processing MS-MS data. DynamX (v. 3.0, Waters) was used for processing HDX-MS data.

For manuscripts utilizing custom algorithms or software that are central to the research but not yet described in published literature, software must be made available to editors and reviewers. We strongly encourage code deposition in a community repository (e.g. GitHub). See the Nature Portfolio guidelines for submitting code & software for further information.

Data

Policy information about availability of data

All manuscripts must include a data availability statement. This statement should provide the following information, where applicable:

- Accession codes, unique identifiers, or web links for publicly available datasets
- A description of any restrictions on data availability
- For clinical datasets or third party data, please ensure that the statement adheres to our policy

To allow access to the HDX data of this study, the HDX summary table (Table S5) and the HDX data tables (Tables S6-S9) are included in the supplementary information, according to the community-based recommendations38. All the deuterium uptake plots of the experiments presented in this work are available on the figshare data repository at https://figshare.com/s/a4940a2f06a34e969d7c

Field-specific reporting

Please select the one below that is the best fit for your research. If you are not sure, read the appropriate sections before making your selection.

- [x] Life sciences
- [ ] Behavioural & social sciences
- [ ] Ecological, evolutionary & environmental sciences

For a reference copy of the document with all sections, see nature.com/documents/nr-reporting-summary-flat.pdf

Life sciences study design

All studies must disclose on these points even when the disclosure is negative.

Sample size	Samples were measured in singlets with selected measuring points being measured in triplicates (at least two time points each data set).
Data exclusions	Peptides were excluded if they were insufficiently fragmented, if their signal-to-noise ratio was not suitable for accurate HDX assessment and/or if the mass error was above 10 ppm. Peptides were excluded from the statistical analysis for calculation of the threshold of significance if not present in triplicate measurement.
Replication	Experiments were performed in technical replicates
Randomization	Samples were injected into the LC-MS system according to the labelling time point (i.e. each labelling time point was analyzed to completion before starting a new time point). Samples were injected in a random order withing the individual time points.
Blinding	Blinding was not relevant for this study. No live subjects were involved and the sample preparations performed in an experimental laboratory.

Reporting for specific materials, systems and methods

We require information from authors about some types of materials, experimental systems and methods used in many studies. Here, indicate whether each material, system or method listed is relevant to your study. If you are not sure if a list item applies to your research, read the appropriate section before selecting a response.

Materials & experimental systems

n/a	Involved in the study	
x		Antibodies
x		Eukaryotic cell lines
x		Palaeontology and archaeology
x		Animals and other organisms
x		Human research participants
x		Clinical data
x		Dual use research of concern

Methods

n/a	Involved in the study	
x		ChIP-seq
x		Flow cytometry
x		MRI-based neuroimaging
